# A solution for highly efficient electroporation of primary cytotoxic T lymphocytes

**DOI:** 10.1186/s12896-024-00839-4

**Published:** 2024-03-26

**Authors:** Nadia Alawar, Claudia Schirra, Meltem Hohmann, Ute Becherer

**Affiliations:** Department of Cellular Neurophysiology, Center for Integrative Physiology and Molecular Medicine (CIPMM), Saarland University, Homburg, 66421 Germany

**Keywords:** CD8^+^ cell, Transfection, Nucleofection, Recovery medium, Viability, Functionality, TIRF, Tetraspanins, Secretion, Plasmid DNA

## Abstract

**Background:**

Cytotoxic T lymphocytes (CTLs) are central players in the adaptive immune response. Their functional characterization and clinical research depend on efficient and reliable transfection. Although various methods have been utilized, electroporation remains the preferred technique for transient gene over-expression. However, the efficiency of electroporation is reduced for human and mouse primary CTLs. Lonza offers kits that effectively improve plasmid DNA transfection quality. Unfortunately, the removal of key components of the cell recovery medium considerably reduced the efficiency of their kit for CTLs. Our aim was to develop a new recovery medium to be used with Lonza’s Nucleofector system that would significantly enhance transfection rates.

**Results:**

We assessed the impact of different media in which the primary CTLs were placed to recover after electroporation on cell survival, transfection rate and their ability to form an immunological synapse and to perform exocytosis. We transfected the cells with pmax-GFP and large constructs encoding for either CD81-super ecliptic pHluorin or granzyme B-pHuji. The comparison of five different media for mouse and two for human CTLs demonstrated that our new recovery medium composed of Opti-MEM-GlutaMAX supplemented with HEPES, DMSO and sodium pyruvate gave the best result in cell survival (> 50%) and transfection rate (> 30 and 20% for mouse and human cells, respectively). More importantly, the functionality of CTLs was at least twice as high as with the original Lonza recovery medium. In addition, our RM significantly improved transfection efficacy of natural killer cells that are notoriously hard to electroporate.

**Conclusion:**

Our results show that successful transfection depends not only on the electroporation medium and pulse sequence but also on the medium applied for cell recovery. In addition, we have reduced our reliance on proprietary products by designing an effective recovery medium for both mouse and human primary CTLs and other lymphocytes that can be easily implemented by any laboratory. We expect that this recovery medium will have a significant impact on both fundamental and applied research in immunology.

## Background

Cytotoxic T lymphocytes (CTLs) and natural killer (NK) cells are at the forefront of combating cancer and viral infections. To do so, they recognize specific structures on the target cell, either through their T cell receptor for CTLs or via various receptors for NK cells. They engage with the target cell by forming an immunological synapse (IS). They polarize a large number of organelles, including the endoplasmic reticulum, mitochondria, and lytic granules (LG), to the IS. A rapid increase in calcium triggers exocytosis of the LG, resulting in the release of proteases like granzymes (Gzm), granulysin, and perforin, which mediate the target cell’s death [1–3]. All of these processes are tightly regulated, and any defect in proteins involved in any of these steps can result in severe immunodeficiency, such as hemophagocytic lymphohistiocytosis and familial hemophagocytic lymphohistiocytosis [4, 5]. Therefore, understanding the function of T cells is of utmost importance.

Studying the molecular mechanism of CTLs function requires the manipulation of protein expression, which can be done by gene deletion, overexpression or mutation. In mouse cells, this can be performed by using costly KO animal models and the results have to be proven by reintroducing the missing gene in so called rescue experiments. In human cells, protein function can only be studied by observing the effect of gene over-expression or gene knock-down by siRNA or KO by CRISPR/Cas9. These gene manipulations require a well-functioning transfection system but many cells are difficult to transfect including primary CTLs [6]. Three methods can be used to transfect CTLs. Chemical transfection using lipofectamine has been used but did not yield a high transfection efficiency [7]. The second one encompasses the use of viruses especially lentivirus as a transfection vector. The results are not consistent [8–10]. Lentivirus integration into the genome is a prerequisite for effective protein expression, making it a preferred method for long-term transfection and stable cell line generation. Notably, gene integration can potentially lead to dysfunctional expression or cell toxicity, depending on its location in the genome [11]. For the transient transfection of CTLs, gene integration is detrimental because it occurs over approximately 1 week during which the cells divide and mature. In addition, high-quality pseudoviral particle preparation and transduction are labor-intensive, time-consuming procedures requiring extensive technical knowledge and biosafety level 2 laboratories. Finally, transfection using lentivirus poses limits to the size of the transfected gene to about 4 kb [12] (maximum plasmid size is 8.5 kb whereby 4.5 kb are occupied by required resistance marker, promoter and regulatory elements). These challenges have led researchers to investigate non-viral transfection methods, such as the electroporation of the cells with DNA or RNA encoding the protein of choice. Electroporation is a faster and cheaper method to transfect cells since no virus particles have to be generated. Additionally, it is safer as no integration in the genome is required minimizing off-target effects. However, this method is impeded with low survival rates thereby reducing the functionality of the transfected CTLs.

The basis of electroporation is the temporary disruption of the cell membrane caused by exposure to an electric field, which permits the entry of molecules into the cell. The formation of pores in the plasma membrane renders the cells fragile requiring a well-adapted protocol so that the plasma membrane reseals quickly. Essential for the success of electroporation is the selection of appropriate buffers used during the electroporation and the media used to recover cells post-electroporation to enhance cell viability [13, 14]. Another challenge with this method lies in the wide range of protocols available for the electrical pulses applied to the cells, which can differ in shape, strength, duration and repetition. As a result, achieving optimal transfection conditions can be a daunting task. Companies like Lonza, with its Nucleofector system, and ThermoFisher, with its Neon system, offer key-in-hand solutions. Their package includes a machine with a preloaded transfection protocol that delivers distinct sequences of electroporation pulses and buffers that are tuned for specific cell types. Several reports have shown great results in transfecting primary T cells using Lonza Nucleofector machines [15, 16]. However, the recovery medium required when using the Nucleofector™ 2b Device to transfect murine CTL was recently withdrawn from the market and a specific recovery media required for human CTL transfection with the 4D-Nucleofector® X Unit was never recommended.

The company suggests alternative approaches such as the use of RPMI medium for transfection of primary T cells. This medium allows the transfection of either small constructs, such as GFP in CTLs, or the transfection of other cell types. However, the outcomes for transfecting constructs encoding larger proteins like pcDNA-GzmB-pHuji and pmax-CD81-SEP (5.441 kb and 4.312 kb, respectively) in CTLs were totally ineffective. We have developed a novel recovery medium (RM) for the Lonza Nucleofector systems that significantly increases the viability and transfection efficiency of primary murine and human CTLs by over three-fold compared to Lonza’s recommended RPMI recovery media. Furthermore, we showed with a functional assay that our new RM strongly promoted the secretory activity of the CTLs. Finally, we provide a detailed transfection protocol that will simplify the work of everyone investigating mouse and human CTLs cell biology.

## Materials and methods

### Mouse cytotoxic T cell preparation

All experimental procedures were approved and performed according to the regulations by the state of Saarland (Landesamt für Verbraucherschutz, AZ.: 2.4.1.1). Mice (C57BL6/N, Charles River Laboratories) of both sexes were housed at 22 °C room temperature with 50–60% humidity and subjected to a standard 12-hour light/dark cycle. They were kept under specific pathogen-free (SPF) conditions and provided with food and water ad libitum. CO_2_ was used to anesthetize the mice (AVMA Guidelines 2007), followed by cervical dislocation to euthanize the animal. Splenocytes were isolated from 13 to 22 weeks old C57/BL6N mice as described previously [17]. Briefly, CD8^+^ T cells were positively isolated from splenocytes of WT mice using Dynabeads Flowcomp CD8 positive isolation kit (Invitrogen) following manufacturer’s instructions. The naïve CD8^+^ T cells were activated with anti-CD3/anti-CD28 coated activator beads (ratio 1:0.8) and cultured for 5 days at 37 °C with 5% CO_2_. The resulting CTLs were cultured at a density 10^6^ cells/ml in 24 well plates with AIM-V medium (Invitrogen) containing 10% fetal calf serum (FCS), 100 U/ml recombinant IL-2 (Gibco), 50 μM 2-mercaptoethanol.

### Human cell culture

Ethical Approval: Research with human PBMC has been approved by the local ethic committee (98/15; Dr. Krause). We obtained the leukocyte reduction system (LRS) chambers, a by-product of platelet collection from healthy blood donors, from the local blood bank (Institute of Clinical Hemostaseology and Transfusion Medicine, Saarland University Medical Center). All blood donors provided written consent to use their blood for research purposes.

Human PBLs were obtained from healthy donors as previously described [18]. Naïve CD8^+^ T cells were isolated using the Dynabeads™ untouched human CD8^+^ T-cell isolation kit (Invitrogen), stimulated with human CD3/CD28 activator beads (Thermo Fisher Scientific) and cultured for 5 days in AIM-V medium supplemented with 10% FCS and 100 U/ml of recombinant human IL-2 (Gibco).

Primary human NK cells were negatively isolated from PBMC using Dynabeads® Untouched™ Human NK Cells Kits (Miltenyi biotec) as described previously [19] and then cultured in AIM-V media with 10% FCS in the presence of recombinant human IL-2 (100 U/ml, Miltenyi) for 2 days.

Untouched CD4^+^ T cells were isolated from PBMC using a CD4 negative isolation kit (Dynabeads Untouched Human CD4 T Cells; Thermo Fisher Scientific) and stimulated with Dynabeads Human T-Activator CD3/CD28 (Thermo Fisher Scientific) and 50 U/ml IL-2 [18].

### Plasmids used for transfection

The human pcDNA-GzmB-pHuji construct was generated by replacing the mTFP at the C-terminus of pcDNA-GzmB-mTFP [20] with pHuji using a forward primer that included an AgeI restriction site 5′-ATG TAT ATC CAC CGG TCG CCA CCA TGG TGA GCA AGG GCG AGG AG-3′ and a reverse primer that included a NotI restriction site 5′-AAG GAA AAA AGC GGC CGC TTA CTT GTA CAG GTC-3′. The size of this plasmid was 5.441 kb. The pmax-CD81-super ecliptic pHluorin (SEP) was generated by subcloning CD81-SEP from pCMV-CD81-SEP, a generous gift from Frederik Verweij (Centre de Psychiatrie et neurosciences, Amsterdam/Paris) into pMax with the restriction sites EcoRI and XbaI. Its size was 4.312 kb. All plasmids were confirmed by sequencing with respective forward and reverse primers by Microsynth Seqlab. The pmax-GFP plasmid (Lonza, Cologne, Germany) encoding green fluorescent protein (GFP) was used.

### Electroporation

Electroporation was performed on day 5 mouse CTLs using the Nucleofector™ 2b Device (Lonza). About 6 × 10^6^ cells were resuspended with 2 μg plasmid DNA in 100 μl electroporation buffer (Mouse T Cell Nucleofector Kit, VPA-1006; Lonza). The mixture was then transferred to electroporation cuvettes. Electroporation was immediately performed using the special pulse (X-001) for mouse CTLs. The transfected cells were then directly transferred to 3 ml prewarmed recovery media and kept in 12 well plates at 32 °C with 5% CO_2_. After 12 hours of transfection, cells were washed and cultured in AIM-V with 10% FCS at 37 °C and used for experiments 14–18 hours post-electroporation. Different types of recovery medium were used to culture cells following electroporation to determine their impact on cell viability, transfection efficiency, and secretion. These recovery media are 1. RPMI (Invitrogen) with 10% FCS; 2. Opti-MEM-GlutaMAX (ThermoFisher, #51985–026) with 10% FCS; 3. RPMI with 10% FCS and former Lonza component A and B; 4. the formerly commercially available complete Lonza recovery media including component A and B (Mouse T Cell Nucleofector Kit, VPA-1006; Lonza) supplemented with 10% FCS and GlutaMAX; and 5. our in-house recovery media consisting of Opti-MEM-GlutaMAX (ThermoFisher, #51985–026) with 10% FCS, 10 mM HEPES (Invitrogen), 1 mM sodium pyruvate, and 1% DMSO.

Electroporation was performed on day 5 human CTLs using the P3 Primary Cell 4D-Nucleofector X Kit (V4XP-3024; Lonza). About 6 × 10^6^ cells were resuspended with 1.5 μg plasmid DNA in 100 μl electroporation buffer. The mixture was then transferred to electroporation cuvettes and electroporation was immediately performed using the EO-115 program. Cells were then directly transferred to 3 ml prewarmed recovery media. For human cells we compared the following recovery media: 1. AIM-V with 10% FCS and 2. our in-house recovery media (RM) mentioned above. Cells were incubated for 12 hours at 32 °C with 5% CO_2_, then washed with AIM-V with 10% FCS and cultured at 37 °C until measurement.

NK cells were electroporated 2 days after isolation. 5 × 10^6^ NK cells were nucleofected with pmax-GFP Vector (2 μg) using the P3 Primary Cell 4D-Nucleofector Kit (Lonza). Electroporation was performed using the 4D-Nucleofector (Lonza) with the EH-115 program as it appeared to be the most promising based on [21]. The cells were then placed in AIM-V with 10% FCS or our RM for 12 hours at 32 °C. Cells were then used for flow cytometry analysis.

CD4^+^ cells were electroporated 3 days after isolation. Stimulation beads were removed, and 5 × 10^6^ CD4^+^ T cells were nucleofected with pmax-GFP Vector (2 μg) using the P3 Primary Cell 4D-Nucleofector Kit (Lonza). Electroporation was performed using the 4D-Nucleofector (Lonza) with the EO-115 program. Cells were then placed in AIM-V with 10% FCS or our RM for 12 hours at 32 °C. Cells were then used for flow cytometry analysis.

### Flow cytometry

About 3 × 10^6^ cells were collected and centrifuged for 6 min at 300 x g and washed with D-PBS (Gibco). The pellet was resuspended in 400 μl D-PBS (Gibco) and used for flow cytometry. The same procedure was performed with 0.2–0.5 × 10^6^ untransfected control cells. Data were acquired by using a BD FACSAriaIII analyzer (BD Biosciences) with BD FACSDivaTM software. Transfection was measured with the FITC channel whereas survival was analyzed based on granularity. The data were analyzed with FlowJo v10.0.7 software. All analysis was performed as two technical replicates. For murine CTL we used 3 different cultures (i.e. three different mice) whereas the human cells came from one donor.

### Total internal reflection fluorescence microscopy (TIRFM)

The TIRFM setup from Visitron Systems GmbH (Puchheim, Germany) was based on an IX83 (Olympus) equipped with the Olympus autofocus module, a UAPON100XOTIRF NA 1.49 objective (Olympus), a 488 nm 100 mW laser, a 100 mW solid-state laser emitting at 561 nm, the iLAS2 illumination control system (Roper Scientific SAS, France), the evolve- Prime95B camera (Teledyne Photometrics) and a filter cube containing Semrock (Rochester, NY, USA) FF444/520/590/Di01 dichroic and FF01–465/537/623 emission filter. The setup was controlled by Visiview software (Version:4.0.0.11, Visitron GmbH). Human CTLs were electroporated with a GzmB-pHuji plasmid as a cytotoxic granule marker, and mouse CTLs were electroporated with CD81-SEP as a multivesicular body (MVB) marker. The functional assay consisted in imaging the fusion of the labeled vesicles with TIRFM. To this end, 14–18 hours after transfection, 0.3 × 10^6^ CTLs were resuspended in 30 μl of extracellular buffer (2 mM HEPES, 140 mM NaCl, 4.5 mM KCl, and 2 mM MgCl_2_) containing no Ca^2+^ and allowed to settle for 1–2 min on anti-CD3 antibodies (30 μg/ml, anti-CD3ε for mouse cells (clone 145-2C11, BD Pharmingen) and anti-CD3 (clone UCHT1, Biolegend) for human cells) coated coverslips. Cells were then perfused with extracellular buffer containing 10 mM Ca^2+^ to promote vesicle fusion. Cells were imaged for 10 min at room temperature either at 561 nm or at 488 nm. The images were taken with an acquisition frequency of 10 Hz and an exposure time of 100 ms. Secretion was analyzed using Fiji V1.4.

### Analysis of transfected cells

Fourteen to eighteen hours after electroporation, cells were assessed for viability based on their morphological appearance in the bright field of TIRFM after IS formation. Nicely attached cells with a clear smooth membrane were considered alive. In contrast, cells with distorted membrane, fragmented and condensed nuclei and visible debris were considered dead. Viability percentage was calculated as number of alive cells × 100 / total number of cells. Transfection efficiency was calculated within each group as the number of transfected cells × 100 / number of alive cells. The number of counted cells was more than 500 per culture. Secretion percentage was calculated as the number of secreted cells × 100 / number of transfected cells.

### Data analysis

To avoid bias in the analysis, data generated to compare cell viability, transfection efficiency and secretion are originating from the same cultures. Data were tested for significance with SigmaPlot (Systat Software Inc.) using Student T test, one-way ANOVA, Mann-Whitney-Test, Kruskall-Wallis-Test with a Dunn’s multiple comparisons post-test: * *p* < 0.05, ** *p* < 0.01, *** *p* < 0.001.

## Results

We performed transfection of murine and human CTLs using the Lonza Nucleofector systems and large constructs that are difficult to transfect. The transfection medium itself was included in the kit and used as described by the company. In this study, we tested various recovery media that are applied to the cells immediately after electroporation. We used the recommended RPMI medium. Further, we used Opti-MEM-GlutaMAX as it has been described to improve transfection using lipofectamine and electroporation of bovine primary fibroblasts [22, 23]. We also combined RPMI with the former Lonza components A and B and the complete Lonza recovery medium encompassing their proprietary media and components A and B. Finally, we used the Opti-MEM-GlutaMAX supplemented with 10 mM HEPES, 1% DMSO and 1 mM sodium pyruvate (new RM). All these media contained 10% FCS. The rationale behind choosing our supplements was the following: The HEPES stabilizes the pH, DMSO supports membrane recovery and sodium pyruvate reduces reactive oxygen species (ROS).

### New recovery medium restores cell viability to the level of the former Lonza medium

**Fig. 1:**
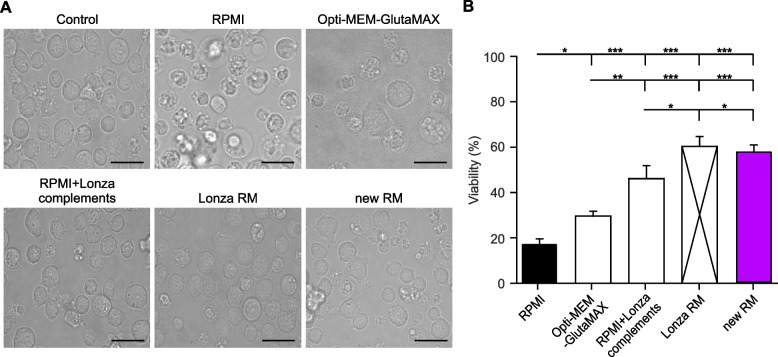
Effect of recovery media on cell viability post-electroporation. **A** Representative brightfield images of primary mouse CTLs after transfection with CD81-SEP in comparison to non-electroporated control cells. Cells were cultivated post-transfection in indicated recovery media for 12 hours at 32 °C and transferred to AIM-V medium for additional 2 to 6 hours at 37 °C before measurements. Lonza RM corresponds to Lonza’s proprietary media complemented with substances A and B. New RM corresponds to Opti-MEM-GlutaMAX supplemented with 10 mM HEPES, 1% DMSO and 1 mM sodium pyruvate. All media contained 10% FCS. Scale bar: 5 μm. **B** Quantitative analysis of cell viability 14 hours post-electroporation using indicated recovery media. Cell viability was determined by measuring the percentage of live cells relative to the total number of cells. Data represents mean ± standard error of the mean (SEM). *N* = 4 independent experiments, *p < 0.05, **p < 0.01, *** *p* < 0.001

Cell viability is a critical parameter in the context of electroporation, as it directly impacts the functionality of primary cells. We tested how the recovery media that is used to culture cells post-electroporation is affecting it. Primary mouse CTLs were subjected to electroporation with 2 μg of CD81-SEP using Lonza 2D nucleofector machine. The electroporated cells were left to recover overnight at 32 °C in five different recovery media including RPMI, Opti-MEM-GlutaMAX, RPMI with Lonza supplements, Lonza RM and our newly developed in-house RM. Comparison between the tested recovery media revealed substantial variations in cell viability. Figure 1 shows that recovering cells in the medium recommended by Lonza namely RPMI yielded the lowest cell viability of 17.35% for mouse CTLs. Other media such as Opti-MEM-GlutaMAX only marginally increased the survival of the cells. A significant increase in cell viability was observed when adding former Lonza components A and B to RPMI, showing the importance of enriching the media with supplements that could help in rescuing cells post-electroporation. Notably, our new RM and the complete Lonza RM that is no longer available showed the highest cell viabilities of 58.19 and 60.33% respectively (Fig. 1B).

**Fig. 2:**
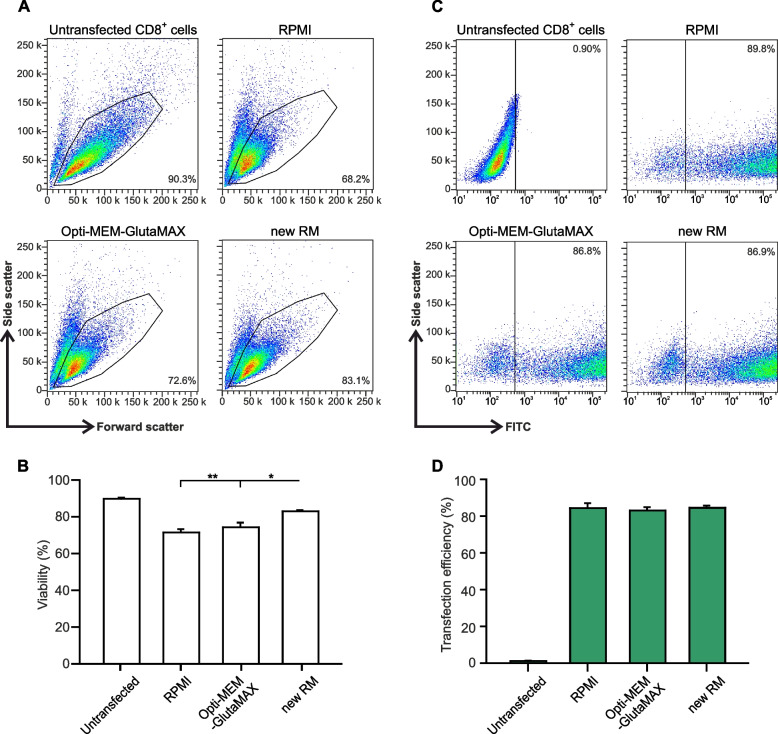
Cell viability of mouse CTLs electroporated with pmax-GFP was increased using new recovery medium. **A** Representative flow cytometry analysis of cell viability of primary mouse CTLs after electroporation. Cells were transfected with pmax-GFP and kept to recover in the indicated recovery media for 12 hours at 32 °C. Viable cells were gated based on forward and side scatter. Cell viability was normalized to control (untransfected cells). Numbers in plots represent the percentage of viable cells in the gate. **B** Quantitative analysis of cell viability 12 hours post-electroporation using the indicated recovery media. **C** Representative flow cytometry analysis of transfection efficiency 12 hours post-electroporation. Transfection efficiency corresponds to the percentage of GFP-positive cells relative to the percentage of viable cells in each condition. Numbers in plots represent the percentage of GFP-positive cells in the gate. **D** Quantitative analysis of the transfection efficiency measured 12 hours after electroporation. Data represents mean ± standard error of the mean (SEM). *N* = 3, *p < 0.05, **p < 0.01

To be able to compare our results to previous studies, we also used the pmax-GFP Vector provided with the transfection Kit to transfect mouse CTLs. The cell viability measured by flow cytometry analysis was similar when cells recovered from transfection in RPMI or Opti-MEM-GlutaMAX. In contrast, our RM outperformed both media showing about 10% increase in viability (Fig. 2A, B). We conclude that even with a small construct that are easier to electroporate, the RM impacts cell viability.

### Primary mouse cytotoxic T cells show highest transfection efficiency in our new recovery medium

**Fig. 3:**
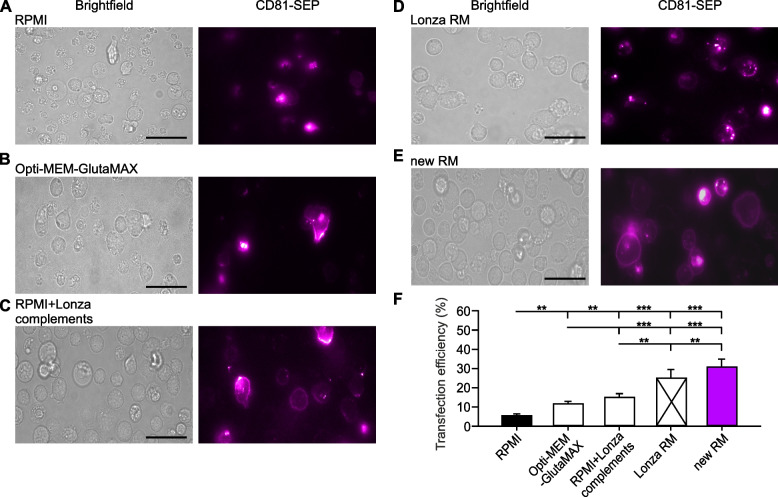
Transfection efficiency of mouse CTLs is maximal in our new recovery medium. **A** to **E**. Representative brightfield (left) and corresponding TIRFM (right) images of primary mouse CTLs electroporated with CD81-SEP and cultured as described in Fig. 1. CTLs were seeded on anti-CD3ε coated coverslips to form an immunological synapse. Images were captured 10 min after seeding. Scale bar: 5 μm. Shown are the result of (**A**) RPMI, (**B**) Opti-MEM-GlutaMAX, (**C**) RPMI with Lonza’s complements A and B, (**D**) Lonza’s complete RM and (**E**) our new RM. **F** Quantitative analysis of transfection efficiency expressed as the percentage of fluorescently labeled cells relative to the total number of viable cells. Data represents mean ± SEM. *N* = 4, *p < 0.05, **p < 0.01, *** *p* < 0.001

Transfection efficiency is a key determinant of the success of electroporation-based genetic manipulations. We measured the transfection efficiency of the pmax-GFP Vector by flow cytometry analysis. The proportion of transfected cells was 84% with RPMI as RM. Using Opti-MEM-GlutaMAX or our new RM did not raise the transfection rate (Fig. 2C, D). This shows that with a small construct the maximum achievable transfection rate is easily obtained and cannot be further increased. In contrast, the evaluation of transfection efficiency of our large CD81-SEP construct in various recovery media revealed significant differences. For transfected cells, the use of a basic recovery medium without supplements proved to be unfavorable (Fig. 3). Our results show the lowest transfection efficiency of 5.78% using RPMI alone for the recovery of transfected cells. When RPMI was enriched with the former Lonza supplements, the transfection efficiency increased significantly by a factor of three. Intriguingly, using Opti-MEM-GlutaMAX without supplement resulted in comparable transfection efficiency as RPMI with supplements. This indicates that the supplements by themself are not sufficient to achieve satisfactory transfection conditions. However, combining Opti-MEM-GlutaMAX with our supplements in the new RM doubled the proportion of transfected cells to 31.02% (Fig. 3F). The transfection efficiency with our new RM was at least as good as the no longer available Lonza mouse CTL RM (Fig. 3F). Altogether our results show that our RM not only improves cell viability of primary mouse T cells post-electroporation, but also contributes to a high transfection rate.

### Primary mouse cytotoxic T cell function improves significantly after recovery in our new RM compared to the former Lonza medium

**Fig. 4:**
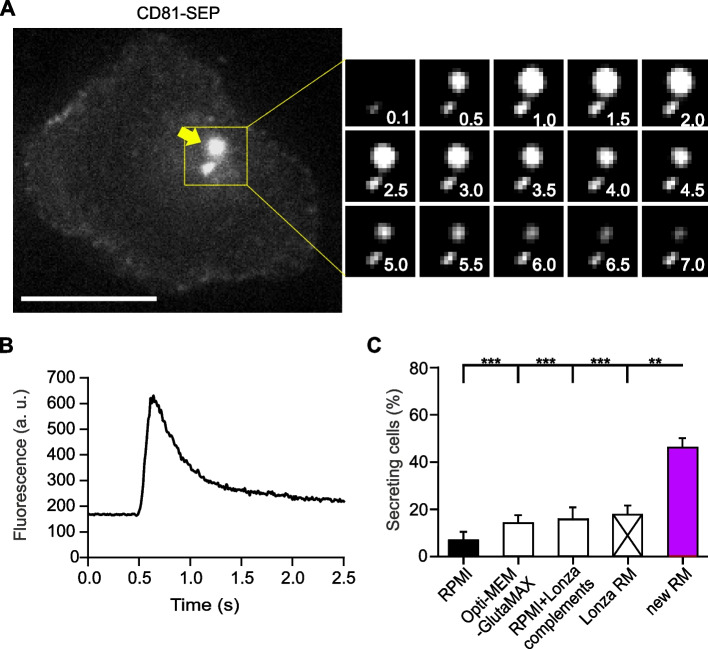
Our new recovery medium surpasses all other media in promoting the function of primary mouse CTLs. **A** Total internal reflection fluorescence microscopy (TIRFM) image of a mouse CTL transfected with CD81-SEP and cultivated with our new RM following the method described in Fig. 1. CTLs were seeded on anti-CD3ε coated coverslips to form an immunological synapse and to induce secretion, which is monitored for 10 minutes at 10 Hz. The yellow arrow shows a multivesicular body. Its fusion is displayed as a sequence of enlarged snapshots on the right. The time points of the displayed snapshots are indicated in seconds. Scale bar: 5 μm. **B** Fluorescent intensity variation during exocytosis of the multivesicular body indicated with the yellow arrow in (**A**). **C** Quantitative analysis of the proportion of secreting CTLs indicated as percentage of secreting cells relative to the total number of transfected cells. Data represents mean ± SEM. *N* = 4 independent experiments, *n* = 31, 57, 20, 36, 19 cells in the order of RM displayed on the graph, *p < 0.05, **p < 0.01, *** *p* < 0.001

The successful expression of the transfected protein and the survival of the CTLs after electroporation is essential. However, it is equally important that they retain their function. The main function of CTLs is the IS formation followed by the release of cytotoxic substances upon target cell contact. In addition, CTLs release exosomes and supramolecular attack particles (SMAPs) that are important in the regulation of the immune response [2, 24, 25]. To evaluate the functional competence of the transfected primary mouse CTLs, we overexpressed the exosome marker CD81-SEP and measured the exocytosis of CD81-SEP-positive vesicles (i.e. multivesicular bodies (MVB)) with TIRFM. For that purpose, 16 ± 2 hours after electroporation, we seeded the CTLs on anti-CD3ε antibody coated coverslips to stimulate exosome release [24]. The fusion of MVB is visualized as a sharp increase of fluorescence as the fusion pore opens and the lumen of the vesicle containing CD81-SEP labelled exosomes deprotonate, followed by a fast loss of the fluorescence as the exosomes diffuse out of the MVB (Fig. 4A, B). We compared the effect of the different recovery media components on cell secretion. The RM had no effect on the number of secreted CD81-SEP labeled vesicles as we observed 2.2 ± 0.3 events per cell in every RM. However, the RM had a strong impact on the proportion of secreting cells. Correlating with the low cell viability and transfection efficiency when using basic RPMI or Opti-MEM-GlutaMAX as recovery media (Figs. 1B and 3F), we observed a reduced number of secreting cells in these RMs (Fig. 4C). The addition of Lonza supplements to RPMI induced a slight improvement of their number reaching the same level of cells maintained in Lonza RM. Electroporated mouse CTLs that were left to recover in our new RM showed by far the highest percentage of functional cells. We recorded more than 45% of secreting cells for this RM, which is 2.5 times higher than in comparison to the proprietary Lonza RM (Fig. 4C).

Our findings demonstrate that the enhanced cell viability and transfection efficiency seen during the recovery of primary mouse T cells in our RM post-electroporation further transferred into improved cell function as indicated by a significantly higher proportion of secreting cells.

### The new recovery medium compared to recommended AIM-V boost human CTL survival and transfection rate by more than seven times allowing measurement of lytic granule exocytosis

**Fig. 5:**
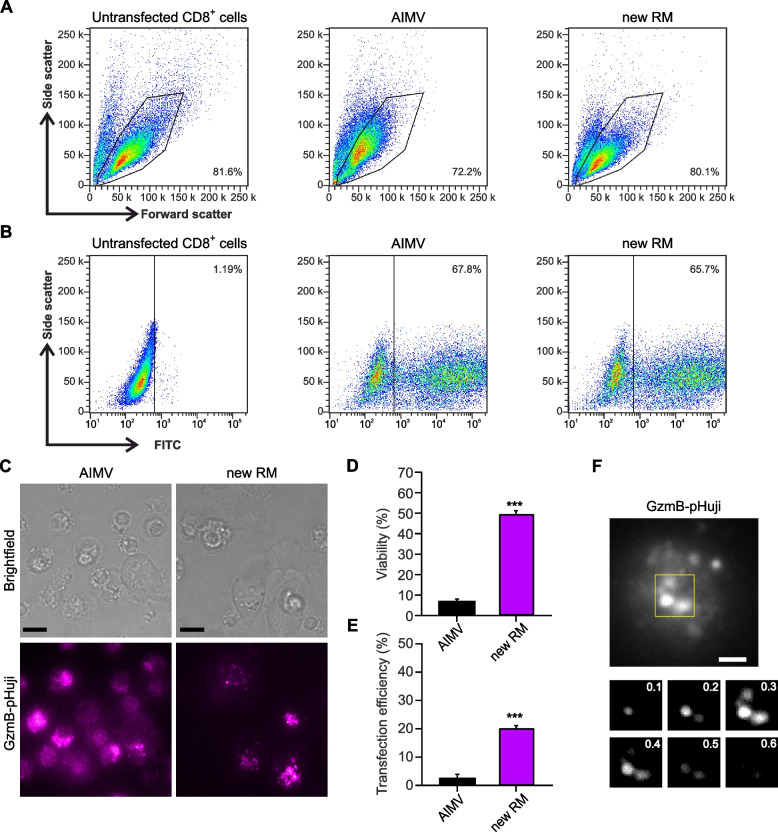
Effect of recovery media on cell viability, transfection efficiency and secretion in human CTLs. **A**, **B**. Representative flow cytometry analysis of cell viability and transfection efficiency of primary human CTLs 12 hours after electroporation with pmax-GFP. Human CTLs were allowed to recover for 12 hours post-electroporation at 32 °C either in AIM-V (middle) or our new RM (right) and compared to untransfected cells (left). **A** Viable cells were gated based on forward and side scatter. Cell viability was normalized to control (untransfected cells). Numbers in plots represent the percentage of viable cells in the gate. **B** Transfection efficiency corresponds to the percentage of GFP-positive cells relative to the percentage of viable cells in each condition. Numbers in plots represent the percentage of GFP-positive cells in the gate. **C-F** represent the results obtained from human CTL transfected with GzmB-pHuji. Cells were recovering from electroporation for 12 hours either in AIM-V or our new RM at 32 °C before being placed for an additional 2 hours in fresh AIM-V at 37 °C. **C**. Representative brightfield images (top) and corresponding GzmB-pHuji images (bottom) of the CTLs. To allow immunological synapse formation, cells were seeded on anti-CD3 coated coverslips. Displayed images were acquired 10 minutes after seeding. Scale bar: 5 μm. **D**, **E** Quantitative analysis of cell viability (**D**) and transfection efficiency (**E**) expressed as the percentage of viable cells relative to the total number of cells and the percentage of fluorescently labeled cells relative to the total number of viable cells, respectively. Data represents mean ± SEM. *N* = 4 independent experiments, *** *p* < 0.001. **F** Top: TIRFM image of a human CTL transfected with GzmB-pHuji on anti-CD3 coated glass coverslip showing lytic granule exocytosis. Scale bar: 1 μm. Bottom: Sequential snapshot of one exocytosis event. Upon fusion with the plasma membrane the lytic granule becomes bright as the granule lumen is neutralized through contact with the extracellular medium. Then it quickly loses its fluorescence (in less than 300 ms) as the GzmB-pHuji diffuses away. Images were acquired at 10 Hz. Time points of the displayed snapshots are indicated in seconds

Most gene defects that affect CTL function have been identified in humans. Thus, it is crucial to investigate gene function in primary human CTLs. These cells are even more difficult to electroporate than mouse CTLs. Therefore, it was imperative to translate our findings from mouse CTLs to human CTLs electroporation. AIM-V is a serum free medium recommended by Lonza that is utilized for the recovery of human CTLs following electroporation. We compared the effect of AIM-V media with our new RM on viability, transfection efficacy and functionality of the human CTLs. We first tested transfection with the pmax-GFP Vector and assessed cell viability and transfection rate by flow cytometry. We found that our RM raise the cell survival by 10% (Fig. 5A as compared to cells that recovered in AIM-V, while the transfection efficacy was similar in both media (Fig. 5B). To test the efficacy of our RM with a construct meeting normal experimental conditions, the cells were electroporated with 1.5 μg of GzmB-pHuji to label the lytic granules and analyzed them 16 hours post-electroporation (Fig. 5C). Our results surpassed our expectations (Fig. 5D, E). The cell viability and the transfection efficiency post-electroporation were more than 8-fold higher in our new RM in comparison to AIM-V (Fig. 5B, C). For the functional assay, we monitored the release of GzmB-pHuji with TIRFM after seeding the cells on anti-CD3ε antibody coated coverslips (Fig. 5F). The viability of the cells incubated in the AIM-V as RM was so low that screening for healthy transfected cells was challenging, and measuring any secretion from these cells was nearly impossible. In contrast, cells maintained in our new RM showed such a high percentage of viable and transfected cells that detecting lytic granule secretion from human CTLs was straight-forward. Overall, 46.5 ± 0.6% transfected cells displayed secretory events with an average of 2.4 ± 0.5 events per cell. In conclusion, our new RM not only significantly improved the transfection of mouse CTLs but also that of human CTLs.

### Recovery medium enhances transfection of other hard to transfect cells

**Fig. 6:**
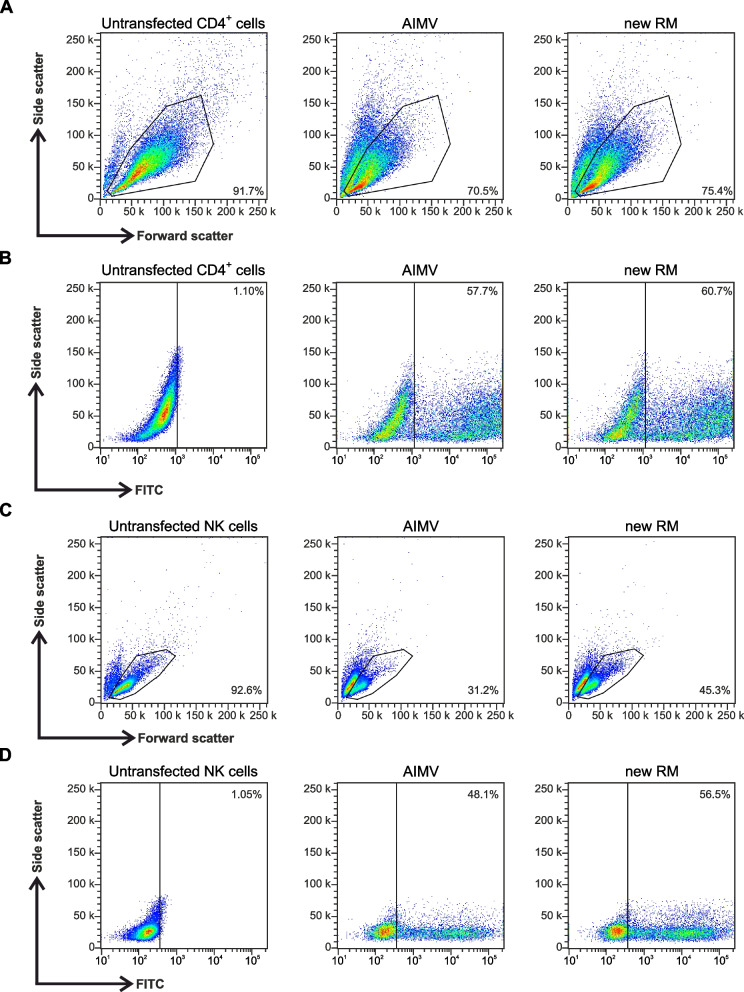
New RM increases natural killer cell viability after transfection. Representative flow cytometry analysis of primary human CD4^+^ cells and natural killer (NK) cells after electroporation with pmax-GFP. Cells were transfected pmax-GFP and cultured for 12 hours at 32 °C either in AIM-V (middle) or our new RM (right). Cell viability was assessed for CD4^+^ cells (**A)** and NK cells (**B**) 12 hours post-electroporation using flow cytometry. Viable cells were gated based on forward and side scatter (left). Viable cells were normalized to control (untransfected cells). Numbers in plots represent the percentage of viable cells in the gate. Transfection efficiency was measured for CD4^+^ cells (**C**) and NK cells (**D**) using flow cytometry 12 hours after electroporation. Transfection efficiency corresponds to the percentage of GFP-positive cells relative to the percentage of viable cells in each condition. Numbers in plots represent the percentage of GFP-positive cells in the gate

We investigated if our new RM could also be used to improve transfection of other hard to transfect cells. For that purpose, we chose human CD4^+^ cells and natural killer (NK) cells. We transfected the cells with the pmax-GFP Vector and assessed cell viability and transfection efficacy through flow cytometry analysis. We compared our RM with AIM-V, which is a culture medium classically used for these cell types [18, 19, 26]. For CD4^+^ cells, our new RM had a limited impact on the survival rate and proportion of transfected cells, both of which increased by approximately 5% (Fig. 6A, B). Regarding NK cells, the survival and transfection rates using AIM-V as the RM were 31 and 48%, respectively (Fig. 6C). Interestingly, the viability increased by 45% while the proportion of transfected cells increased by 17% for NK cells that recovered in our new RM compared to AIM-V (Fig. 6D).

## Discussion

Transfecting primary T cells is essential as it enables the precise manipulation of their genetic and functional characteristics. This provides crucial insights into T cell biology and facilitates the use of T cell therapy for various diseases, including autoimmune disorders and cancer. This study was conducted to overcome the well-known complexity of transfecting primary human and mouse cells. We selected primary CTLs as a representative cell type for difficult-to-be electroporated cells. Commercial electroporation kits, especially those from Lonza, used in this study, proved to be effective. However, discontinuation of key components reduced greatly their efficiency, and the alternatives recommended by the company were not equivalent to the original ingredients. This shows that not only the correct transfection medium is important to obtain a successful transfection with high cell survival, but that the RM is also decisive. Therefore, we conducted a study to determine the optimal composition of an alternative recovery medium that would enhance cell viability, transfection efficiency, and cell function after electroporation. We assessed the impact of RM on the transfection of pmax-GFP Vector to be comparable with other transfection optimization paradigms [21, 22, 27]. Nevertheless, for mouse CTL the highest possible transfection efficacy of more than 85% was reached with the most basic medium. This transfection rate does not leave much room of improvement when testing new media and highlights the limitation of using this pmax-GFP to benchmark a transfection method. Therefore, we opted for larger and more complex vectors that were more challenging to electroporate and that better simulate real life laboratory conditions.

We compared the effects of different basic media on the recovery of electroporated primary mouse CTLs. The choice of media was directed by the following criteria. The medium needed to work well with T-cell culture, making DMEM, commonly used for adherent cells, unsuitable. Appropriate media options included RPMI (as recommended by the company), IMDM, which was unsatisfactory for cell expansion in our experience, or AIM-V that is predominantly used for human CTLs [28, 29]. Opti-MEM-GlutaMAX is recommended by the company for transfection using lipofectamine. Furthermore, several studies showed good results using this media for electroporation [22, 23, 30]. Therefore, we selected Opti-MEM-GlutaMAX as the basis for our new RM and compared it to RPMI. Opti-MEM-GlutaMAX was associated with better cell viability, transfection efficiency, and organelle secretion. We supplemented this medium with 10 mM HEPES to reduce damaging changes in pH after transfection. For better comparison in between media we included 10% FCS in all RM. Additionally, FCS was present in all our culture media and removing it in the RM might have stressed the cells. The transfection efficiency and cell viability were lower when the basic media were used compared to the original Lonza RM with its supplements. Consequently, we decided to augment Opti-MEM-GlutaMAX plus FCS and HEPES with additional supplements.

Dimethyl sulfoxide (DMSO) has been reported to enhance the transfection efficiency and cell viability during and after electroporation in mammalian cells [31]. The use of DMSO seems to be counterintuitive because it is an amphipathic substance used to solubilize water insoluble substances. It permeates freely membranes by forming pores and at high concentration it induces cell death [32]. However, it has been shown that at relatively low concentration it stabilized the impaired cell membrane and increased the survival rate [31]. Thus, we supplemented our basis RM with 1% DMSO. The permeabilization of the cell membrane during electroporation induces cell stress involving a high energy consumption and oxidative stress in cells, resulting in the generation of reactive oxygen species (ROS). To enhance cellular ATP production, we hypothesized that supplying an intermediate metabolite of glycolysis, specifically sodium pyruvate - known also as a ROS scavenger [33] would be advantageous. Notably, sodium pyruvate has been reported to protect cell health whilst promoting growth and survival, thereby indirectly improving transfection efficiency [34]. Thus, we incorporated sodium pyruvate into our RM at a final concentration of 1 mM. Finally, we used transient (12 to 14 hours) mild hypothermic (32 °C) culture conditions to reduce cell stress that is induced by massive over-expression of proteins. Indeed, reduced culture temperature has been used in CHO cells to improve recombinant protein productivity [35, 36].

Our results show that the newly composed RM has not only improved cell viability and transfection efficiency but also had a positive impact on cellular secretion processes. This emphasizes that our optimized RM has a positive effect on physiological cell function. Our functional data in mouse and human CTLs extends beyond the measurement of a single form of secretion. We observed the exocytosis of MVBs, which release exosomes, in mouse CTLs, and the release of granzyme from lytic granules in human CTLs. In mouse CTLs the over threefold increase in transfection efficiency and cell viability between cells that were incubated in RPMI vs. our new RM, leads to an increased proportion of secreting cells. Moreover, we found that the functionality of CTLs incubated in our new RM surpassed that of the former complete Lonza RM. The results were even more dramatic when we used our RM for primary human cells. For these cells no specific RM is known therefore AIM-V medium has been widely used [37]. Previous studies, in which plasmids encoding for larger constructs than GFP were used, display a very low efficiency of electroporation of activated primary human T cells [38]. Similarly, we obtained a transfection efficiency and viability below 1transfection of other lymphocytes. We found that it especially improved the viability0% using the AIM-V as RM. However, by using our RM, we were able to increase both parameters by more than 7-fold, thereby dramatically improving the exocytosis activity from these cells. These findings demonstrate the superiority of our new RM over standard media for the post-electroporation recovery of primary human cells. Finally, we assessed the impact of our new RM on the transfection of other lymphocytes. We found that, in comparison to AIM-V (used here) and NK MACS (Miltenyi Biotec, used in [21], our RM especially improved the viability of NK cells after transfection, which is crucial for proper cell function.

## Conclusions

We herein report the development of a cost-effective recovery medium that is easily reproducible in any laboratory, surpassing the affordability of any other commercially available media. Additional suggestions to replace the transfection medium with Opti-MEM-GlutaMAX as a variant might be possible or a combination with published media [27] will make transfection more effective, easier to implement and independent of product change by the supplier or delivery shortages such as experience during the corona crisis. Importantly, our RM allows successful gene transfer in other hard to transfect cells such as NK cells. We are convinced that this improved electroporation protocol will enable future use in CRISPR-Cas9 technology or siRNA transfection in lymphocytes.

## Data Availability

All original data sets (flow cytometry, TIRFM movies and other imaging results) generated for this study have been deposited in ZENODO repository server (https://zenodo.org/) with the dataset identifier 10.5281/zenodo.10631069. The accession code will be provided by the corresponding author upon reasonable request.

## Abbreviations

ATP: Adenosine triphosphate
CTL: Cytotoxic T lymphocyte
DMSO: Dimethyl sulfoxide
FCS: Fetal calf serum
GFP: Green fluorescent protein
GzmB: Granzyme B
HEPES: 4-(2-hydroxyethyl)-1-piperazineethanesulfonic acid
MVB: Multi vesicular body
NK: Natural killer
RM: Recovery medium
RPMI: Roswell Park Memorial Institute
ROS: Reactive oxygen species
SEP: Super ecliptic pHluorin
SMAPs: Supramolecular attack particles
TIRFM: Total internal reflection fluorescent microscopy
